# The Mechanical, Thermal, and Biological Properties of Materials Intended for Dental Implants: A Comparison of Three Types of Poly(aryl-ether-ketones) (PEEK and PEKK)

**DOI:** 10.3390/polym15183706

**Published:** 2023-09-08

**Authors:** Sandra Paszkiewicz, Paweł Lesiak, Konrad Walkowiak, Izabela Irska, Karol Miądlicki, Marcin Królikowski, Elżbieta Piesowicz, Paweł Figiel

**Affiliations:** 1 Faculty of Mechanical Engineering and Mechatronics, West Pomeranian University of Technology, 70-310 Szczecin, Poland; wk42388@zut.edu.pl (K.W.); izabela.irska@zut.edu.pl (I.I.);; 2 Tele-Fonika Kable S.A., Factory in Bydgoszcz, 85-957 Bydgoszcz, Poland; pawel.lesiak@tfkable.com

**Keywords:** 3D printing, PAEK, dental implants, thermal properties, mechanical properties, biological test

## Abstract

Regarding the dynamic development of 3D printing technology, as well as its application in a growing part of industries, i.e., in the automotive industry, construction industry, medical industry, etc., there is a notable opportunity for its application in producing dental implants, which presents a promising alternative to traditional implant manufacturing methods. The medical industry is very restrictive regarding the applied materials, and it is necessary to use materials that exhibit very good mechanical and thermal parameters, show clinical indifference and biocompatibility, are non-allergenic and non-cancerous, and are likely to sterilize. Such materials are poly(aryl-ether-ketone)s (PAEK)s, mainly poly(ether-ether-ketone) (PEEK) and poly(ether-ketone-ketone) (PEKK), that are found to be high-performance polymers and can be defined as materials that retain their functionality even in extreme conditions. In the present paper, two types of PEEKs and PEKK were compared regarding their structural, mechanical, and thermal properties along with the biological activity toward selected strains. The tested samples were obtained with Fused Deposition Modeling (FDM) technology. The PEKK, after heat treatment, exhibits the most promising mechanical properties as well as less bacterial adhesion on its surface when compared to both PEEKs. Consequently, among the evaluated materials, PEKK after heat treatment stands out as the optimal selection for a dental prosthesis.

## 1. Introduction

Nowadays, 3D printing can be seen in all industries, from the medical industry, automotive and mechanical industry, to the construction industry. This is because, in additive manufacturing (AM), one can obtain prints from various materials and for various applications. Considering the different AM methods such as FDM (Fused Deposition Modeling), SLA (Stereolithography), DLP (Digital Light Processing), or LOM (Laminated Object Manufacturing), etc., with the further development of these methods, one can see that 3D printing is getting more popular and therefore it is used even more often in other industries and not only in the previously mentioned ones. The FDM method uses a thermoplastic polymer filament, which is then heated in a nozzle to a semi-liquid state [1,2]. Then, in a semi-fluid state, it is extruded onto the bed surface or on previously extruded material layers [1]. One of this method’s most important properties is the polymer filament’s thermoplasticity because it allows the filament to melt during printing and solidify after printing [1]. Significant advantages of the FDM method are the low costs of the printer and the material, the simplicity of the method, and the speed of the processes.

According to a market analysis, the 3D printing market was estimated to be worth USD 11.58 billion in 2019 and is expected to expand at a CAGR of more than 14% between 2020 and 2027. Globally, 1.42 million units of 3D printers were shipped in 2018, which is expected to reach 8.04 million units by 2027 [3]. Confirmation of the rapid development of 3D printing can also be seen in the number of patents submitted in particular years [4]. The use of 3D construction printers allows for reducing construction time, construction costs, and waste while increasing accuracy [5].

Furthermore, 3D printing is mainly suitable for prototyping and unit production and has found its application in medicine. Three-dimensional printing is often used in medicine due to the possibility of personalizing dentures thanks to carefully prepared digital models [6]. Thus, it can be translated into better patient treatment results [6]. Recently, 3D printing regarding dental implants has been industrialized and is also more often used for medical devices, surgery planning, and medical education [6]. Considering that currently made implants are made of metals and ceramics, which makes their cost relatively high as a result, polymers from the PAEK family may be a suitable replacement for traditional implants [7,8,9].

The materials from the poly(aryl-ether-ketone) (PAEK) family belong to high-performance polymers. They can be defined as materials that retain functionality even in extreme conditions, i.e., low and high temperatures, have excellent mechanical properties, and have environmental resistance. PEEK is this family’s most well-known material, which perfectly fits the definition of a high-performance polymer. PEEK appeared in the late 1990s as a semi-crystalline material with great mechanical and physical properties, excellent thermal stability, non-allergic properties, and low plaque affinity [10,11]. Due to its excellent biological properties, PEEK is widely used in medicine, e.g., cranial and orthopedic implants, and in a wide range of applications in restorative, prosthetic, and implant dentistry [10].

This study aims to compare two types of PEEKs and PEKK for their structural, mechanical, and thermal properties’ differences along with the biological activity towards selected strains. In addition, the printing conditions for the two PEEKs were determined. Therefore, in the present paper, the filaments are characterized using Fourier-transform–infrared-spectroscopy (FTIR), differential scanning calorimetry (DSC), and a thermogravimetric analysis (TGA). Subsequently, based on the results of the DSC analysis, the initial parameters of the printing process were determined, which were then updated using trial and error, which allowed us to obtain a series of samples with various degrees of infill. The obtained samples (dumbbell-shape samples and bars) were subjected to mechanical tests through tensile, bending, and impact tests. Furthermore, samples with 100% infill were analyzed using X-ray diffraction (XRD). Moreover, since the materials are intended for prosthetic purposes, the biofilm formation on the surface of the 3D printed roll was assessed using a qualitative method (Richards).

## 2. Materials and Methods

### 2.1. Materials and Preparation of the Samples

Three poly(aryl-ether-ketones) were selected for the test by means of the following:

1. A PEEK filament bought from 3DGence (Katowice, Poland), with a diameter of 1.75 mm, coded as “PEEK 1”;
2. A PEEK-nature filament bought from W2 Filaments (Kepfenberg, Austria), with a diameter of 1.75 mm, coded as “PEEK 2”;
3. A Kimya PEKK-A filament by Arkema (Colombes, France), with a diameter of 1.75 mm, coded as PEKK.

The information provided by materials’ manufacturers on the mechanical properties is summarized in Table 1.

### 2.2. Characterization Methods

Attenuated total-reflectance–Fourier-Transform-Infrared (ATR-FTIR) spectra taken from the filaments were recorded using an FTIR spectrophotometer ThermoNicolet iS5 apparatus (Thermo Fisher Scientific, Waltham, MA, USA) in the range of frequency of 400–4000 cm^−1^. Each material was scanned 17 times.

The samples’ structure was analyzed with a differential scanning calorimeter (DSC). Measurements were carried out with a DSC 204 F1 Phoenix (Netzsch, Selb, Germany) at a heating–cooling–heating cycle of 10 °C/min, in the temperature range of 50–400 °C. The first cooling and second heating scans were used to determine the melting and crystallization peaks. Subsequently, from the second heating cycle, the glass transition temperature (T_g_) was estimated as the midpoint of the change in heat capacity. The following equation (Equation (1)) determined the degree of crystallinity:

(1) $$X_{c}=\frac{∆H_{m}}{∆H_{m}^{0}}\cdot 100\%$$

where ΔH_m_ is the enthalpy of melting derived from the melting peak area on DSC thermograms, and ΔH_m0_ is the enthalpy of melting for fully crystalline material (130 J/g for PEEK [15] and 130 J/g for PEKK [13].

Moreover, using a DSC analysis, the heat treatment procedure for PEKK was conducted to present the changes in the material during annealing (heat treatment) in a vacuum oven (Binder VDL-23, Tuttlingen, Germany). The procedure was conducted following the manufacturer’s recommendations: (i) fast cooling (20 °C/min) to 160 °C; (ii) annealing at 160 °C for 30 min; (iii) slow heating (1 °C/min), reflecting the heating rate of the material in the dryer; (iv) annealing at 200 °C for 15 min (the observations of the PEKK’s color change); (v) subsequent heating up to 400 °C (10 °C/min); and (vi) annealing at 400 °C for 5 min and finally cooling the material to room temperature.

The thermo-oxidative stability of the filaments used in this study was evaluated with thermogravimetry (TGA 92-16.18 Setaram, Caluire, France) using the system measuring TG-DSC simultaneously. Measurements were carried out in an oxidizing atmosphere (i.e., dry, synthetic air (N_2_:O_2_ = 80:20 vol.%)). The study was conducted at a heating rate of 10 °C/min in the temperature range of 20–700 °C.

Size Exclusion Chromatography (SEC) was performed using polystyrene reference standards. The number average molecular mass (M_n_) and the polydispersity index (PDI = M_w_/M_n_) were evaluated.

The tensile properties of the 3D printed materials were measured according to ISO 527 using an Autograph AG-X plus (Shimadzu, Kyoto, Japan) tensile testing machine (class 1.0 according to EN 10002-2, ISO 7500-1, BS 1610, ASTM E4, and JIS B7721), equipped with a 1 kN Shimadzu load cell, an optical extensometer (class 0.5 according to ISO 9513), and TRAPEZIUM X computer software (version 1.4.5, Shimadzu, Kyoto, Japan), operated at a constant crosshead speed of 1 mm/min. Measurements were performed at room temperature on the dumbbell samples with a grip distance of 30 mm. Seven measurements were conducted for each dumbbell-shaped sample (type A3), and the results were averaged to obtain a mean value.

The flexural properties were measured according to ISO 178 using an Autograph AG-X plus (Shimadzu, Kyoto, Japan) testing machine (class 1.0 according to EN 10002-2, ISO 7500-1, BS 1610, ASTM E4, and JIS B7721), equipped with a 1 kN Shimadzu load cell, operated at a constant crosshead speed of 1 mm/min. Measurements were performed on the samples with the dimensions of l = 80 ± 2 mm, b = 10 ± 0.2 mm, and h = 4 ± 0.2 mm.

The impact strength was determined with the Charpy method according to the standard ISO 179-1/1eU and type of the sample: 1 (l = 80 ± 2 mm, b = 10 ± 0.2 mm, and h = 4 ± 0.2 mm), edge impact (e), and no notch (U). Five measurements were conducted, and the results were averaged to obtain a mean value.

The X-ray diffraction (XRD) analysis was conducted with the use of a Panalytical X’Pert diffractometer (Malvern Panalytical, Malvern, UK) operating at 40 V and 40 mA with CuKα radiation (λ = 0.154 nm). The samples were scanned from 2θ = 4° to 70°, with a step of 0.02°.

In the microbiological study, we used the following reference strains: Staphylococcus aureus ATCC 25923, Escherichia coli ATCC 25922, Pseudomonas aeruginosa ATCC 27853, Enterococcus faecalis ATCC 29212, and Candida albicans ATCC 10231. The Columbia agar medium with 5% sheep blood (bioMérieux, Warsaw, Poland) was used for breeding bacteria. In contrast, the Sabouraud substrate (bioMérieux, Warsaw, Poland) was utilized to cultivate yeast. Incubation was performed at 37 °C for 24 h under aerobic conditions. The investigation of the formation of biofilm on the surface of prosthetic materials was conducted with the quality method. The study of the formation of bacterial and fungal biofilms on the surface of prosthetic materials cut to 1:1 cm was estimated using a qualitative method—Richards. A suspension with a density of 1.0 on the McFarland scale was prepared from a 24 h culture grown on a TSB medium, and then sterile specimens of prosthetic materials were placed inside. After a 24 h incubation at 37 °C, the samples were washed three times with NaCl, and 1 drop of a 1% 2,3,5-triphenyltetrazaliium chloride (TTC) solution was added. The samples were again subjected to a 24 h incubation at 37 °C. All tests were carried out in duplicate.

### 2.3. Preparation of the Samples

**Figure 1:**
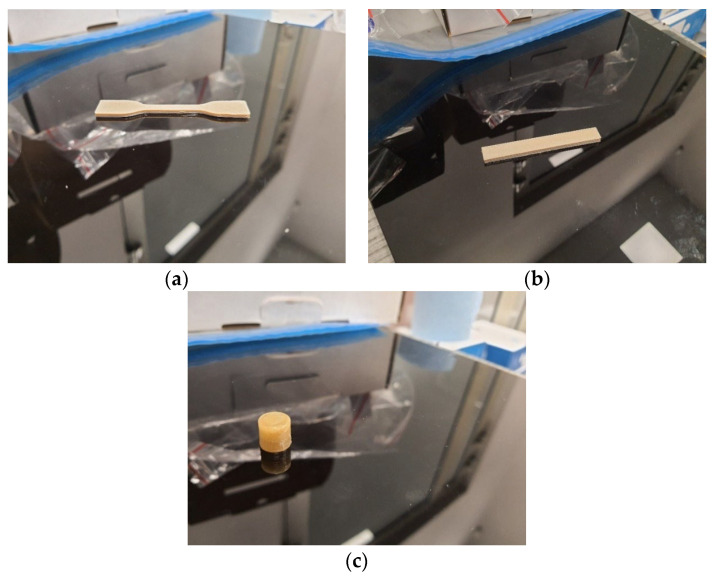
The appearance of printed samples: dumbbell-shaped samples (type A3) (**a**); samples for the flexural properties and the impact strength (**b**); and the sample of a cylindrical shape for biological research (**c**).

All samples were prepared using the FDM method on an INTAMSYS Funmat HT 3D printer (Shanghai, China). To carry out the tensile tests, dumbbell-shaped samples (type ISO 37 type A3) were used; for the flexural properties and the impact strength, samples with the dimensions of l = 80 ± 2 mm, b = 10 ± 0.2 mm, and h = 4 ± 0.2 mm were used; for biological research, non-standard samples of a cylindrical shape with dimensions of d = 10 mm and l = 10 mm were used. The appearance of printed samples is presented in Figure 1.

Despite the existence of recommended printing parameters provided by the manufacturers, the quality of the prints using the suggested parameters was incorrect by means of layer separation and splitting, blobs, zits, and elephant’s foot. Therefore, with the trial and error method, we had to determine the printout parameters to obtain the proper print quality (Table 2). The build plate was cleared each time the process was set up. The selection of the build plate and chamber temperature and the number of layers of the adhesive specimen was partially selected based on the instructions of adhesive specimen manufacturers.

**Figure 2:**
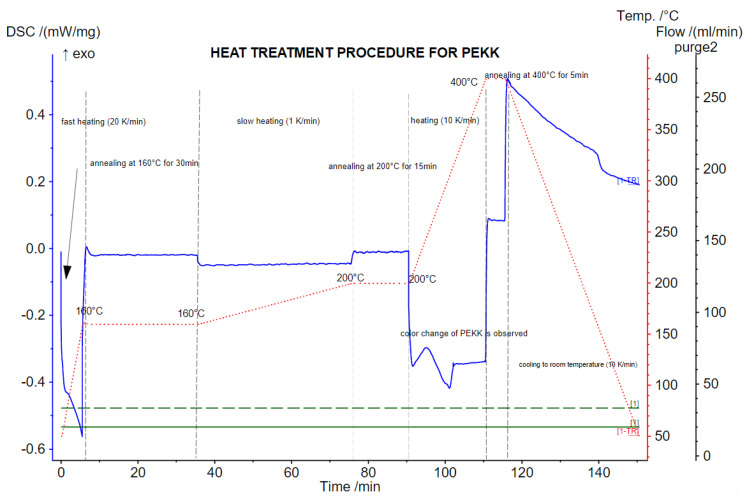
DSC chart reflecting the actual procedure of PEKK heat treatment.

However, it was taken into account that, according to the manufacturer and the literature data [16], annealing of PEKK should result in better mechanical properties. For this reason, PEKK samples were annealed following the recommended procedure presented on the DSC chart (Figure 2), which reflects the actual method of heating PEKK in a vacuum dryer (Binder). These samples were coded as “PEEK HT”.

**Figure 3:**
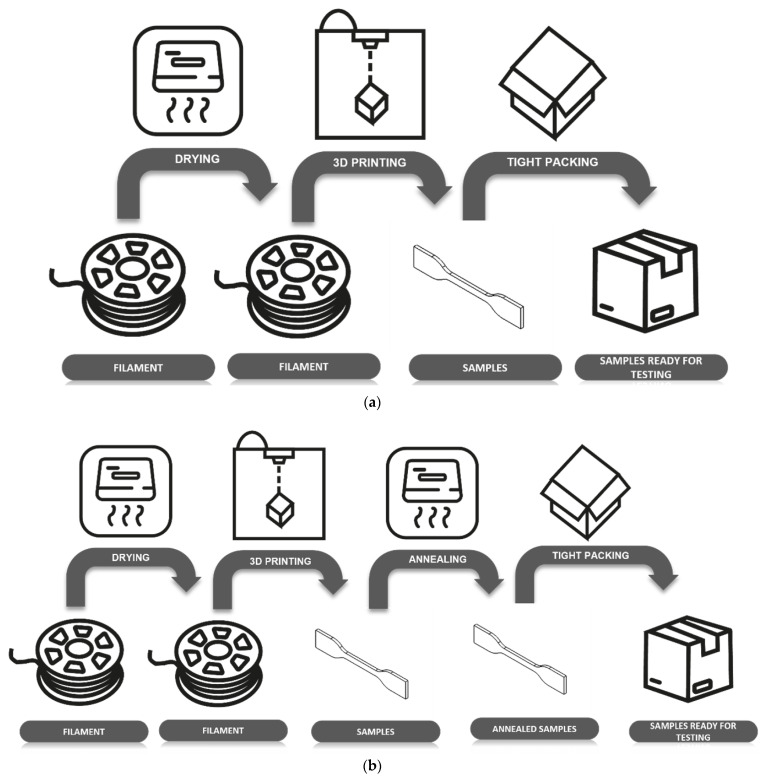
(**a**) Sample preparation process for PEEK 1, PEEK 2, and PEKK. (**b**) Sample preparation process for PEKK HT.

The annealing process began at a temperature of 160 °C, where the sample was then held for 30 min. Subsequently, by heating at 1 °C/min, the temperature rose to 200 °C (heating rate analogous to that in the dryer). At the temperature of 200 °C, the sample was held for 15 min until the color of the sample was uniform. The sample was removed from the dryer and placed in an airtight container. The entire process of preparing samples for testing is shown in Figure 3.

## 3. Results and Discussion

### 3.1. Characterization of Filaments

#### 3.1.1. Fourier Transform Infrared Spectroscopy (FTIR)

**Figure 4:**
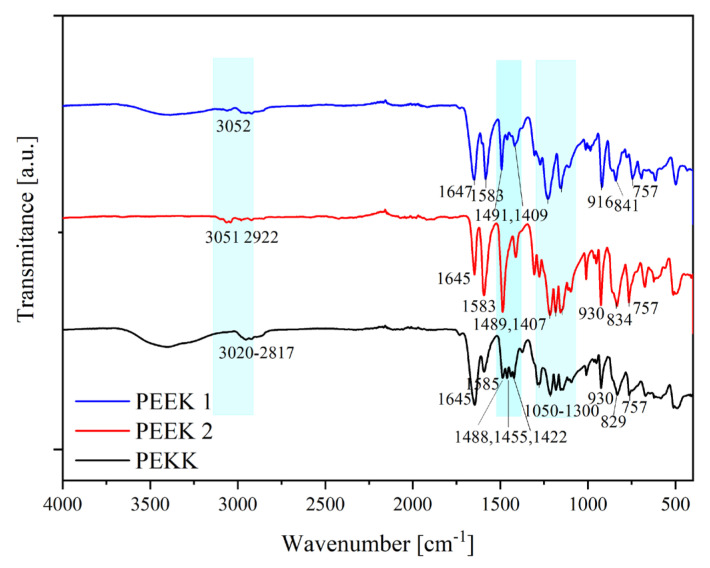
FTIR spectra of two types of PEEKs and PEKK.

To compare two types of PEEKs (PEEK 1 and PEEK 2) and PEKK, FTIR spectroscopy was used. The FTIR spectra of the samples are shown in Figure 4. In addition, the assignments of the FTIR peaks of the analyzed materials are summarized in Table 3. We observed the distinct differences between the FTIR spectra of PEEKs and PEKK. However, both PEEKs differ slightly from one another. We observed a difference in the 1300–1050 cm^−1^ range corresponding to the diphenyl ether group (C-O-C) rotation and stretching [17,18]. However, we observed slight differences between PEEK 1 and PEEK 2 for the remaining peaks. It is, however, different in the case of PEKK. Here, the differences resulting from a different chemical structure are visible (a ketone moiety between benzene rings instead of an oxygen bridge). First of all, differences were observed between the materials in the range of 1300–1050 cm^−1^ and, unlike for both PEEKs, three distinct peaks were observed at 1488 cm^−1^, 1455 cm^−1,^ and 1422 cm^−1^, then two peaks at 1489 cm^−1^ and 1407 cm^−1^ (1409 cm^−1^) for PEEKs [17,19]. These peaks were assigned to the diphenyl ether group (C-O-C) rotation and stretching and aromatic rotations [17,19]. The differences in aromatic rotations, much less the ease of rotation for PEKK than for PEEK, especially confirm the differences between the materials.

#### 3.1.2. Differential Scanning Calorimetry (DSC)

**Figure 5:**
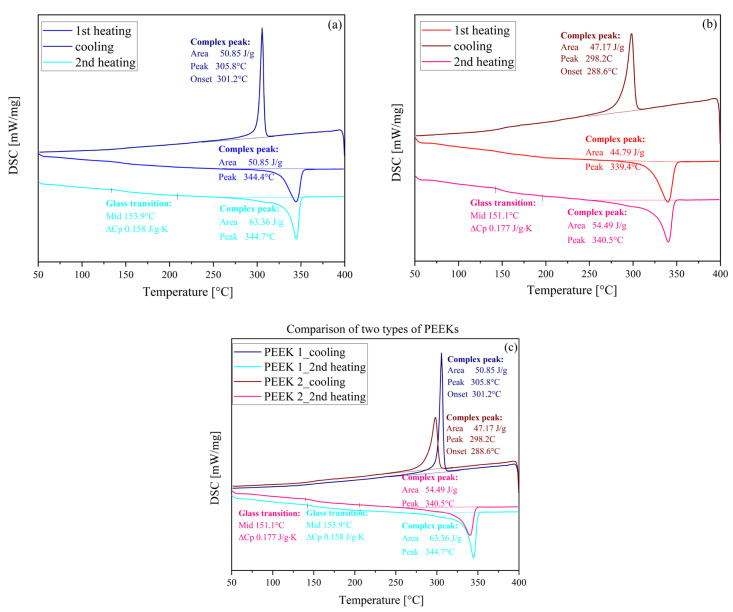
DSC thermograms for PEEK 1 (**a**) and PEEK 2 (**b**), and DSC thermograms comparing the phase transition temperatures for both PEEKs (**c**).

**Figure 6:**
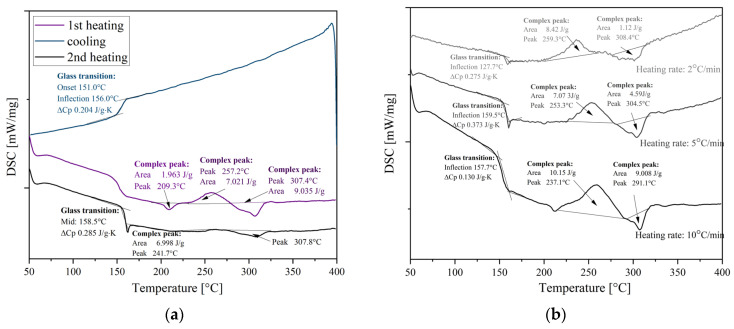
(**a**) DSC thermograms for PEKK at the heating/cooling rate of 10 °C/min; (**b**) DSC thermograms recorded at the second heating scans at different heating rates.

The DSC thermograms of the samples are shown in Figure 5 and Figure 6. Moreover, Figure 6b presents the thermal behavior of PEKK at different heating rates. In the presented DSC thermograms, the first heating curve was not analyzed as it presents the thermal history of the material, and it was only used to erase previous thermal history by heating the material above melting, where relaxation or molecular rearrangement can occur, then cooling at a known rate before heating again (the second heating scan). When comparing both PEEKs (Figure 5), it is clearly visible that PEEK 1 exhibits higher values of all phase transition temperatures, i.e., T_g_, T_m_, and T_c_, than PEEK 2 of ca. 3–4 °C. Moreover, the peaks corresponding to T_c_ and T_m_ are sharper for PEEK 1 (Figure 5c), which indicates a less defected crystalline structure of this polymer [20]. In addition, the values of the degree of crystallinity (estimated based on the ΔH_m_ from the second heating scan) for PEEK 1 (48.7%) are greater than for PEEK 2 (41.9%). In turn, PEKK was less crystalline and exhibited only cold crystallization at different heating rates and no crystallization from the melt. The estimated value of the degree of crystallinity for PEKK equals 5.4% (at a heating rate of 10 °C/min), which clearly demonstrates its less crystalline vulnerability. Furthermore, when comparing the crystallization behavior of PEKK at different heating rates (10, 5, and 2 °C/min), it was found that along with an increase in the heating rate, the decrease in the value of melting temperature and increase in the ΔH_m_ was observed. In addition, a shift toward higher temperature in the case of glass transition was detected at higher heating rates, which might result from the relaxation effects. In addition, the values of the degree of crystallinity at different heating rates were as follows: for 10 °C/min, it was 6.9%; for 5 °C/min, it was 3.5%; while for 2 °C/min, it was 0.86% (which clearly indicates the amorphous behavior of PEKK).

#### 3.1.3. Thermogravimetric Analysis (TGA)

**Figure 7:**
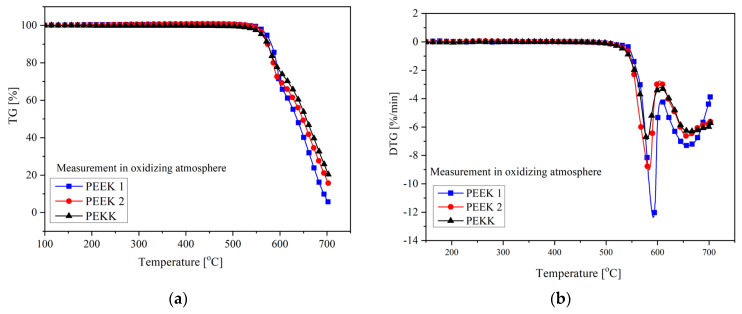
Mass loss (TG) (**a**) and derivative of mass loss (DTG) (**b**) as a function of temperature for PEEK 1, PEEK 2, and PEKK.

An important factor determining the suitability of materials for thermal processing is their thermal stability, especially thermo-oxidative stability. TGA measured the thermo-oxidative stability of the analyzed materials, as shown in Figure 7. The characteristic temperatures for mass losses of 5%, 10%, 50%, and 90%, as well as the temperatures corresponding to the maximum of mass losses, are listed in Table 4. The thermal degradation of all analyzed materials occurs in two steps. In the first decomposition step, random chain scission of the ether and ketone bonds is the main mechanism, with phenol being the predominant degradation product and smaller amounts of other compounds like benzene and dibenzofuran [21,22]. However, cleavage of the carbonyl bond leads to radical intermediates that are more stable due to resonance effects and would be expected to predominate [21,22]. In the second decomposition stage, slower volatilization of the residue occurs, wherein in the employed temperature range of 20–700 °C, this carbonaceous residue was for PEEK 1 ca. 6%, for PEEK 2 ca. 15%, and for PEKK ca. 20%. All analyzed materials’ onset temperatures (5% of mass losses) were observed above 500 °C. The highest value of T_5%_ was observed for PEEK 1, while the lowest was observed for PEKK (a difference of 9 °C). Similar observations were made at 50% of mass loss. However, PEKK proved to be more thermally stable at higher temperatures (above 600 °C) along with the temperature increase. This results probably from the two decomposition mechanisms, in the case of PEEK and PEKK. This is also reflected in the observations on the DTG curve (Figure 7b), where the values of T_DGT1_ are higher for PEEK 1 and PEEK 2, while the value of T_DTG2_ is the highest for PEKK.

### 3.2. Characterization of 3D Printed Samples

#### 3.2.1. Mechanical Properties and Structure of 3D Printed Materials

**Figure 8:**
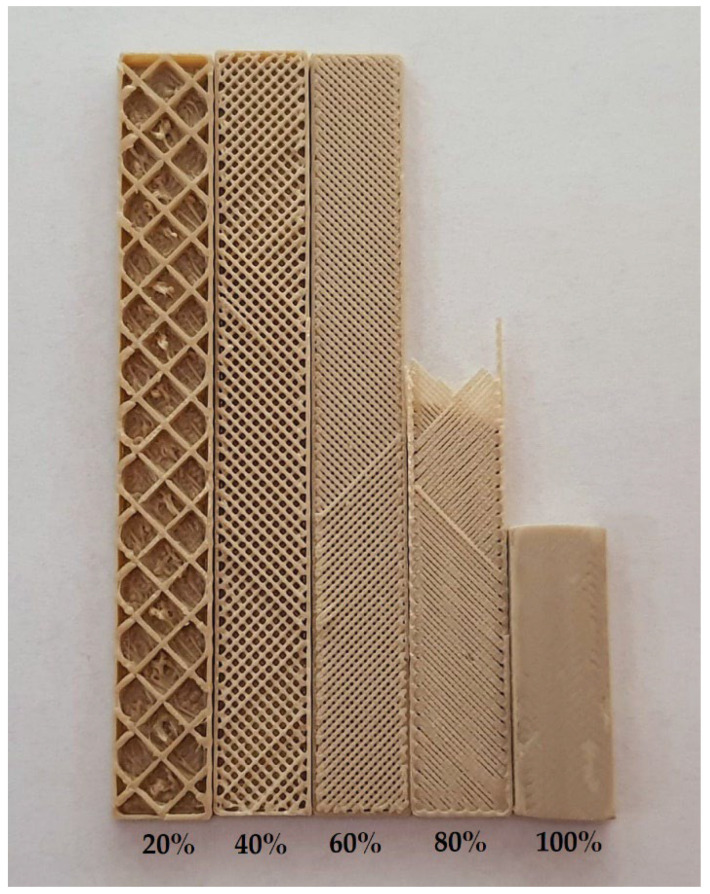
Sample cross-section for the flexural properties and the impact strength, and the appearance of individual infill percentages.

The samples for tensile measurements were prepared with a different percent of infill (from 100 to 20%). However, only samples with 100% infill were tested due to the too small cross-section of the samples with lower infill (20%, 40%, 60%, and 80%), evidenced with the appearance of the samples presented in Figure 8.

**Figure 9:**
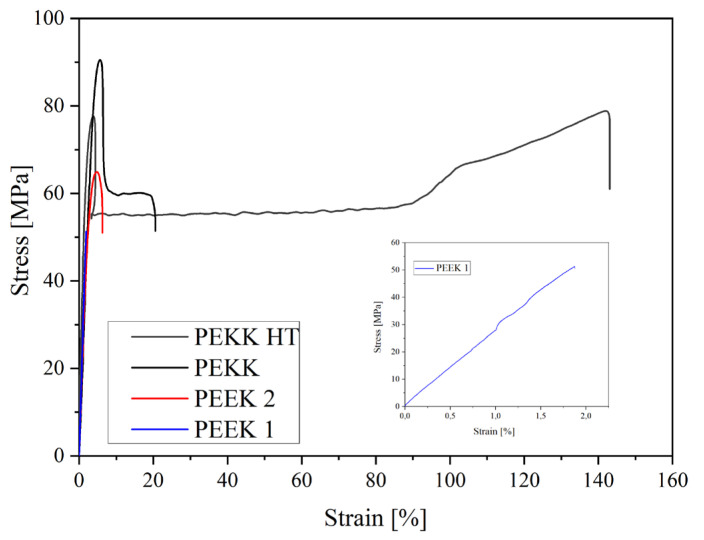
Representative stress–strain curves of static tensile test for printed materials.

Figure 9 shows representative stress–strain curves for PEEK 1, PEEK 2, PEKK, and PEKK HT at 100% infill. The values of the number average molecular weight (M_n_), polydispersity index (PDI), tensile modulus (E), tensile strength at a break (σ_b_), and elongation at a break (ε_b_) are summarized in Table 5. The material with the highest value of M_n_ is PEEK 2, and its value is around 10,000 g/mol higher compared to PEEK 1. M_n_ and PDI values are not mentioned for PEKK HT because it is not affected by heat treatment, so these values are the same as for PEKK. Moreover, one can observe a higher PDI of PEEK 1 compared to PEEK 2. When comparing two PEEKs with each other, it can be seen that PEEK 2 exhibits much better mechanical performance.

**Figure 10:**
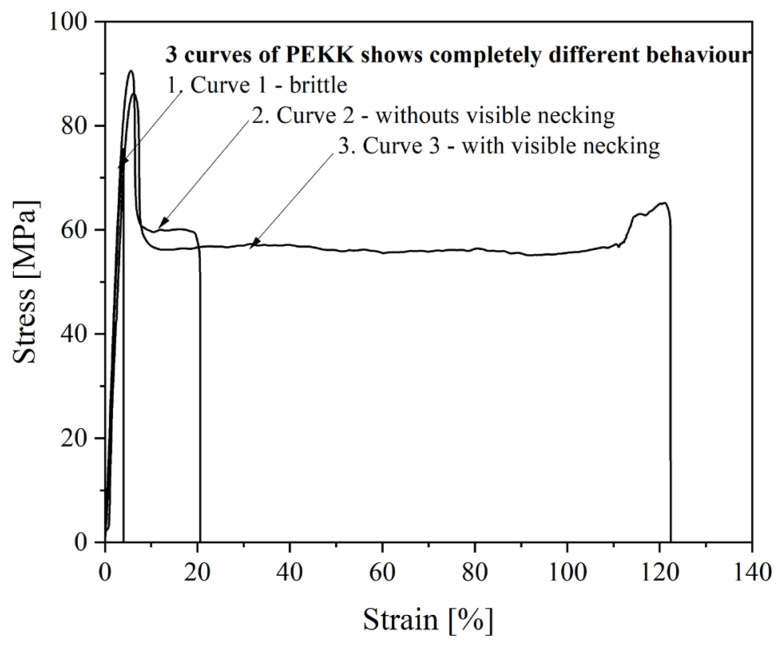
Stress–strain curves showing different material behavior of PEKK.

**Figure 11:**
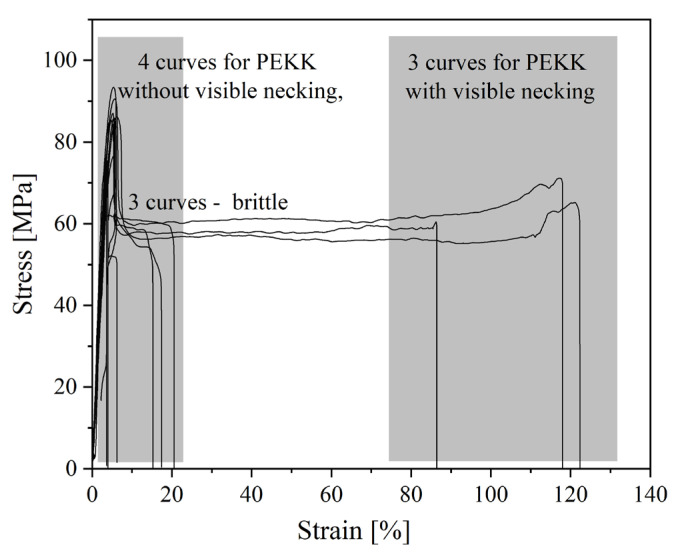
Stress–strain curves of static tensile test for PEKK.

**Figure 12:**
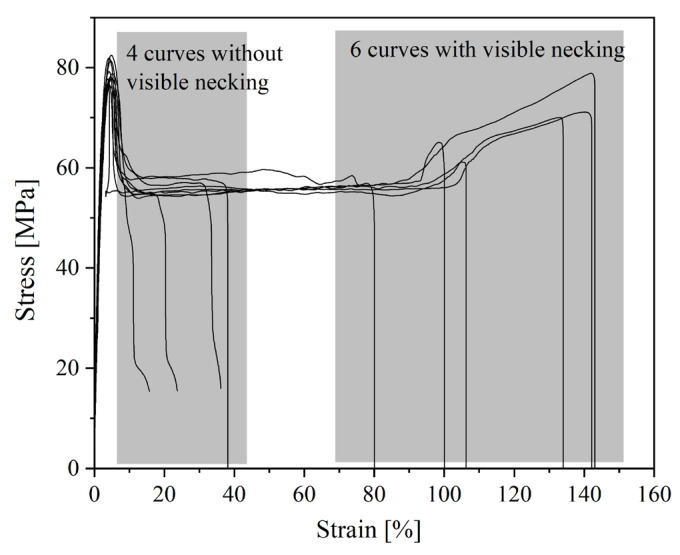
Stress–strain curves of static tensile test for PEKK HT.

**Figure 13:**
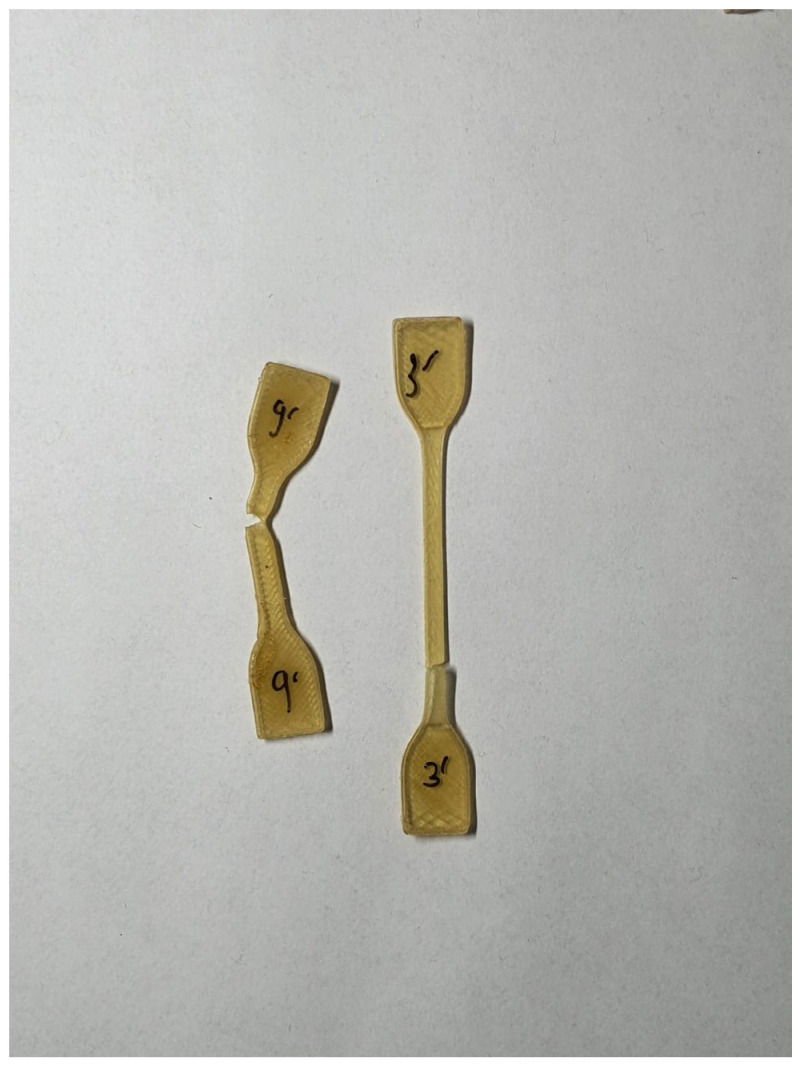
Demonstration of the different behavior of the tested samples during tensile strength.

Nevertheless, both PEEKs are brittle materials. The value of elongation at a break for PEEK 1 is about 2%, while for PEEK 2, it is around 5%. PEEK 2 has a higher value of Young’s modulus and stress at the break than PEEK 1, about 123 MPa and 18 MPa, respectively. The better mechanical performance of PEEK 2 than PEEK 1 is likely due to the higher molecular weight of PEEK 2 [23]. Furthermore, one could observe different behavior for PEKK and PEKK HT. The differences within the series of 3D printed PEKK (10 dumbbell-shape samples) are presented in Figure 10 and Figure 11. It is seen (Figure 11) that materials exhibit different behavior types—brittle, materials without visible necking, and materials with visible necking—which resulted in high values of standard deviation. In addition, Figure 11 shows how many samples in the series exhibited the specified behavior and found that almost equal numbers of samples exhibited the listed behaviors. Moreover, when comparing Figure 12 and Figure 13, it is seen that the heat treatment caused the unification of the structure of the samples, relief of the residual stress, and elimination of internal defects and structural distortions [24,25]. Therefore, the PEKK HT samples did not exhibit brittle behavior and showed higher values of Young’s modulus and elongation at a break compared to PEKK, about 3381 MPa and 42%, respectively. However, heat treatment did not affect the value of stress at a break, which resulted in insignificant differences in stress at break values between PEKK and PEKK HT samples. The results obtained for all the materials indicate their possible use as dental implants [26]. Compared to traditional dental implant materials such as titanium and its alloys, both PEEKs and PEKK exhibit a distinct advantage: their Young’s modulus values are much more closely aligned with those of natural bone, enamel, and dentin [10]. Moreover, the high values of Young’s modulus of titanium and its alloys result in severe stress shielding and failure [27].

**Figure 14:**
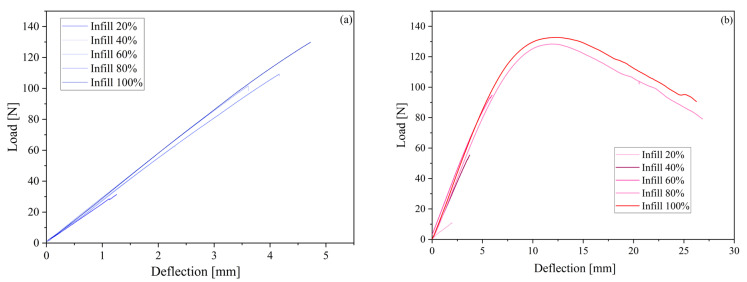

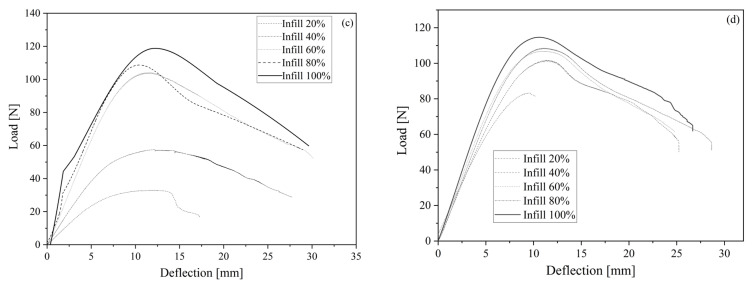
Load–deflection curves for PEEK 1 (**a**), PEEK 2 (**b**), PEKK (**c**), and PEKK HT (**d**).

**Figure 15:**
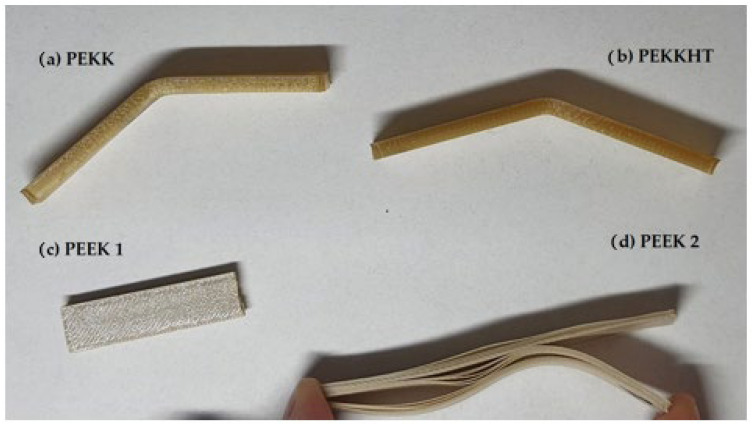
The appearance of the samples after bending.

Figure 14 shows the representative bending curves, whereas Figure 15 shows the appearance of the samples. The relation between load and deflection for PEEK 1 is almost linear. The maximum load for PEEK 1 with 100% infill is 125 N. However, one can observe that compared to PEEK 2, PEKK, and PEKK HT, the deflection value is significantly smaller in the case of PEEK 1, up to 25 mm. As for PEEK 2, the materials at 100% infill have a similar maximum load value than at the infill of 80%. Moreover, samples of PEEK 2 were not as brittle as those printed from PEEK 1 because they were delaminated between the layers, which may prove a low cohesion force. Comparing PEKK and PEKK HT with each other, it can be seen that the heat treatment process unified the structure inside the printout because all infills in PEKK HT show similar behavior to the sample with a 100% infill concentration. Considering the results, it can be concluded that the best properties are obtained at 100% or 80% infill. In contrast, a further decrease in infill caused a significant decrease in the maximum load and deflection value.

**Figure 16:**
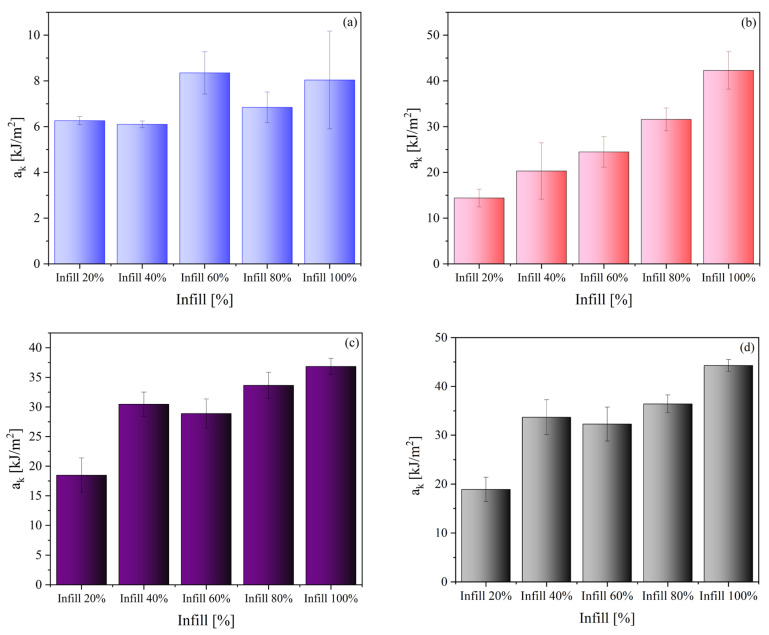
Impact test for PEEK 1 (**a**), PEEK 2 (**b**), PEKK (**c**), and PEKK HT (**d**).

Moreover, impact tests were employed to examine the toughness of the materials, which is a crucial determinant of their capacity to absorb energy while undergoing plastic deformation. Brittle materials have low toughness due to the small amount of plastic deformation they can endure. Figure 16 shows the a_k_ (notch impact strength) values as a function of infill for PEEK 1, PEEK 2, PEKK, and PEKK HT, respectively. The infill concentration had no significant effect on the value of a_k_ for PEEK 1. In turn, for PEEK 2, the values of impact strength increase along with infill concentration; the difference between the lowest concentration (20%) and the highest (100%) was about 27 kJ/m^2^. The values of a_k_ for PEEK 2 were significantly higher than for PEEK 1; the difference between the samples with the highest infill concentration is about 34 kJ/m^2^. While for both PEKKs only in the case of 20% infill, the impact strength values were much lower than for the samples with a higher concentration of infill. The remaining samples based on PEKK within series, with an infill concentration above 40%, exhibited similar behavior, and the differences were insignificant. Moreover, the value of a_k_ was slightly higher for the PEKK HT samples when compared to PEKK. PEKK (before and after heat treatment) generally exhibited higher resistance toward impact force among tested materials.

**Figure 17:**
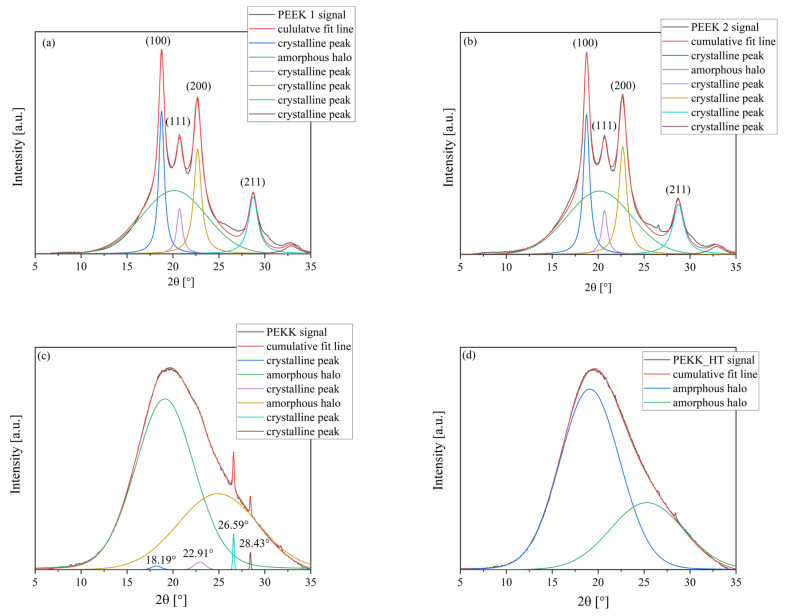
X-ray diffraction (XRD) curves of PEEK 1 (**a**), PEEK 2 (**b**), PEKK (**c**), and PEKK HT (**d**).

Figure 17 shows diffraction patterns for the tested materials. The degree of crystallinity was calculated and is summarized in Table 6. PEEK 1 and PEEK 2 have very similar, almost identical WAXS patterns. Thus, one can conclude that both PEEKs are semi-crystalline thermoplastic polymers and did not exhibit any polymorphism, which agrees with [23]. The three peaks around 2θ at 20° can be assigned to the (110), (111), and (200) planes of crystallized PEEK, respectively, while around 29° corresponds to the (211) plane [28]. Moreover, both PEEKs have a similar degree of crystallinity. Figure 17 presents the WAXS diffractograms of two PEKKs. According to [29], PEKK can be ascribed with a single amorphous halo, but due to the poor fitting, two amorphous halos were used herein. In PEKK, without heat treatment, one can see several crystalline peaks, which are no longer visible in Figure 17d. Furthermore, PEKK, before and after heat treatment, was found to be amorphous material. These observations are in agreement with the ones obtained from the DSC analysis.

#### 3.2.2. Study of Biofilm Formation on the Surface of 3D Printed Materials

Biofilm formation on the surface of three selected polymers typically used as prosthetic materials, through PEEK 1, PEEK 2, and PEKK, was assessed using a qualitative method (Richards), where the following classification of results was adapted depending on the color of the prosthetic material:

(−)—strain not forming the biofilm (corresponded to the lack of cells)
(+)—strain weakly forming the biofilm (corresponded to 10^3^–10^4^ CFU/mL)
(++)—strain strongly forming the biofilm (corresponded to 10^5^–10^6^ CFU/mL)
(+++)—strain strongly forming the biofilm (corresponded to 10^7^–10^8^ CFU/mL)

On the basis of the conducted research, differences in biofilm estimation were observed in the qualitative method (Table 7). Regarding antibacterial activity, according to [10,30,31], PEKK shows less bacterial adhesion on its surface compared to PEEK. The roughness of the surface is also of great importance, i.e., the rougher the surface, the greater the adhesion of bacteria to the substrate [32]. The confirmation of this phenomenon can be seen in Table 7. One can see that PEKK exhibits much better biostatic properties than PEEKs. Comparing PEEK 1 and PEEK 2, it can be seen that PEEK 2 has a greater affinity towards strains of *C. albicans* and *S. aureus*, which might result from a worse surface quality.

## 4. Conclusions

The tested samples were obtained with FDM technology, which is one of the common 3D printing methods. Three materials from the PAEK family were tested, i.e., two types of poly(ether-ether-ketone) (PEEK) and poly(ether-ketone-ketone) (PEKK). Moreover, PEKK was subjected to heat treatment to investigate how it affects its final properties. FTIR confirmed the difference in chemical structure between materials. The values of phase transition temperatures in PEEK 1 and PEEK 2, determined from DSC, did not show significant differences. In turn, PEKK exhibited a higher value of T_g_, a lower value of T_m,_ and a value of X_c_ ~1% (almost completely amorphous). Moreover, the degree of crystallinity of PEKK before and after heat treatment did not change, and it even lowered from 1.13% to 0.16%. In addition, PEKK HT turned out to be the material with the most promising mechanical properties. The improvement of PEKK’s properties after heat treatment was probably due to the relief of the residual stress and the elimination of internal defects and structural distortions. In addition, PEKK exhibited less bacterial adhesion on its surface when compared to both PEEKs.

## Figures and Tables

**Table 1:** Values of mechanical properties according to manufacturers’ data [12,13,14].

Properties	PEEK 1	PEEK 2	PEKK
Tensile strength	105 MPa	87 MPa	65 MPa
Flexural modulus	3.9 GPa	4.2 GPa	1.66 GPa
Elongation at break	30%	8.3%	5%
Impact strength (Charpy)	14 kJ/m^2^	53 kJ/m^2^	2.5 kJ/m^2^

**Table 2:** Printing parameters for the tested materials.

Material	PEEK 1	PEEK 2	PEKK
Temperature of nozzle (°C)	360	380	340
Temperature of build plate (°C)	140	140	140
Temperature of chamber (°C)	90	90	90
Printing speed (mm/s)	25	15	30
Adhesive specimen	Nano Polymer Adhesive^®^ (one layer)	Nano Polymer Adhesive^®^ (one layer)	Magigoo^®^ (3 layers)
Nozzle diameter (mm)	0.4	0.4	0.4
Bead width (mm)	0.4	0.4	0.4
Layer thickness (mm)	0.2	0.2	0.2

**Table 3:** Assignments of the FTIR peaks of two types of PEEKs and PEEK.

Wavenumber (cm^−1^)	Assignment
3051 (3052)	C=C-H stretch vibration
2922 (2817)	-CH_2_ stretch vibration
1645 (1647)	C=O stretch in ketone
1583	Skeletal in-plane vibration or aromatic ring
1489, 1455, 1422, 1407	Aromatic rotations
1300–1050	Diphenyl ether group, C-O-C rotation, and stretch
930	Aromatic out-of-plane bending
829, 757	C-H out-of-plane bending substitution patterns

**Table 4:** TGA parameters for PEEK 1, PEEK 2, and PEKK in the oxidizing atmosphere.

Material	T_5%_	T_10%_	T_50%_	T_90%_	T_DTG1_	T_DTG2_
	(°C)	(°C)	(°C)	(°C)	(°C)	(°C)
PEEK 1	568	580	634	692	591	660
PEEK 2	562	572	647	−	583	659
PEKK	557	572	655	−	579	666

T_5%_, T_10%_, T_50%_, and T_90%_—temperatures corresponding to 5%, 10%, 50%, and 90% of mass loss; T_DTG1_ and T_DTG2_—the temperatures corresponding to the maximum of mass losses.

**Table 5:** Mechanical properties of printed materials.

Sample	M_n_	PDI	E	σ_b_	ε_b_
	(g/mol)		(MPa)	(MPa)	(%)
PEEK 1	28,200	2.4	2236.2 ± 348.6	48.9 ± 9.6	1.8 ± 0.5
PEEK 2	38,600	2.1	2359.9 ± 334.2	66.2 ± 7.7	5.2 ± 1.4
PEKK	31,300	1.9	1384.7 ± 606.6	84.1 ± 6.1	39.6 ± 49.1
PEKK HT	-	-	4765.5 ± 928.3	79.2 ± 2.1	81.9 ± 50.3

M_n_—number average molecular weight, PDI—polydispersity index, E—Young’s modulus, σ_b_—stress at break, and ε_b_—elongation at break.

**Table 6:** Percentage of individual phases in the tested materials.

	PEEK 1	PEEK 2	PEKK	PEKK HT
Amorphous phase share	52.81%	51.35%	98.87%	99.84%
Crystalline phase share	47.19%	48.65%	1.13%	0.16%

**Table 7:** Classification of biofilm formation for individual strains.

Material	The Strain Used in Biofilm Analysis				
	*C. albicans*	*S. aureus*	*P. aeruginosa*	*E. faecalis*	*E. coli*
PEEK 1	++	++	++	+	+
PEEK 2	+++	+++	++	+	+
PEKK	+	+	+	+	+

## Data Availability

The data presented in this study are available on request from the corresponding author.
